# Molecular basis for *Poria cocos* mushroom polysaccharide used as an antitumour drug in China

**DOI:** 10.1111/jcmm.13564

**Published:** 2018-11-15

**Authors:** Xiulian Li, Yanli He, Pengjiao Zeng, Yong Liu, Meng Zhang, Cui Hao, Hua Wang, Zhihua Lv, Lijuan Zhang

**Affiliations:** ^1^ Medical Systems Biology Center for Complex Diseases Affiliated Hospital of Qingdao University Qingdao China; ^2^ School of Medicine and Pharmacy Ocean University of China Qingdao China

**Keywords:** antitumour, clinical application, pharmacological activities, polysaccharides, *Poria cocos*

## Abstract

*Poria cocos* is an edible medicinal fungus known as “Fuling” in Chinese and has been used as a Chinese traditional medicine for more than two thousand years. Pharmacological studies reveal that polysaccharide is the most abundant substance in *Poria cocos* and has a wide range of biological activities including antitumour, immunomodulation, anti‐inflammation, antioxidation, anti‐ageing, antihepatitis, antidiabetics and anti‐haemorrhagic fever effects. As a result, “*Poria cocos* polysaccharide oral solution” was developed and sold as an over‐the‐counter health supplement since 1970s. In 2015, “Polysaccharidum of *Poria cocos* oral solution” was approved as a drug by Chinese Food and Drug Administration for treating multiple types of cancers, hepatitis and other diseases alone or during chemo‐ or radiation therapy for patients with cancer. In this article, biochemical, preclinical and clinical studies of *Poria cocos* polysaccharide from 72 independent studies during the past 46 years (1970‐2016) based on PubMed, VIP (Chongqing VIP Chinese Scientific Journals Database), CNKI (China National Knowledge Infrastructure) and Wanfang database searches are summarized. The structure, pharmacological effects, clinical efficacy, immunobalancing molecular mechanism and toxicity of *Poria cocos* polysaccharide are deliberated to provide a general picture of *Poria cocos* polysaccharide as a clinically used antitumour drug.

## 1 INTRODUCTION

**Figure 1:**
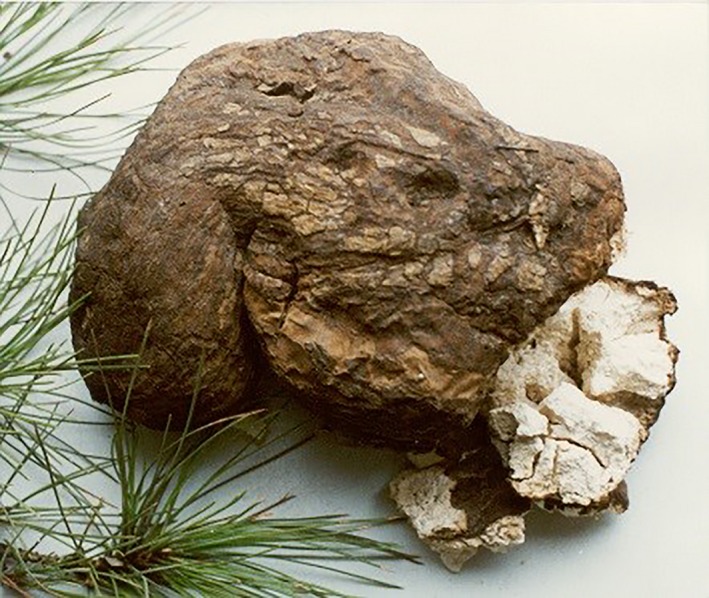
The fruiting body of Mushroom *Poria cocos*. *Poria cocos* is an edible medicinal fungus known as “Fuling” in Chinese and has been used as a Chinese traditional medicine for more than two thousand years

*Poria cocos* (Figure 1), known as “Fuling” in Chinese, is an edible medicinal mushroom belonging to dry sclerotium of polyporaceae fungi. It has more than 2000 years of medical application history for its remarkable pharmaceutical effect.^1^ The bioactive components in *Poria cocos* include polysaccharides, triterpenoids, fatty acids, sterols and enzymes. *Poria cocos* polysaccharide (PCP) accounts for 84% by weight among all constituents in the dried sclerotium.^2^ PCP is also the main bioactive component in *Poria cocos*.

### 1.1 The structural composition and properties of PCP

**Figure 2:**
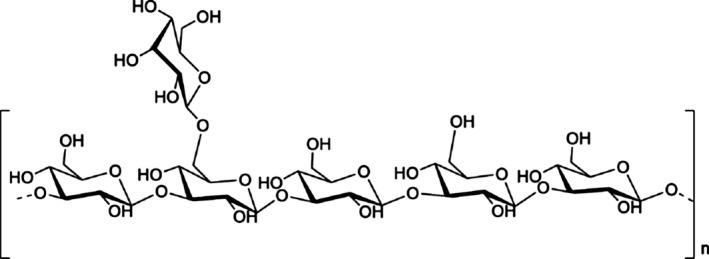
A schematic diagram of β‐glucan structure in *Poria cocos*. β‐Glucan is the major *Poria cocos* polysaccharide with β‐(1→3)‐linked glucose backbone and β‐(1→6)‐linked glucose side chains. The β‐glucan from *Poria cocos* has poor water solubility but decent anticancer activity

PCP is extracted from the sclerotium of *Poria cocos*. Different solvent extraction methods can obtain different polysaccharide fractions, such as WPS (NaOH‐HAc), PAP (1 mol/L NaOH), PCP2 (hot water), PCP1 (0.9% NaCI), PCP3‐I and PCP3‐II (0.5 mol/L NaOH), PCP4‐I and PCP4‐II (88% formic acid).^3^, ^4^ Thus, PCP is a mixture of different types of polysaccharides with the molecular weight ranged from 4.1 × 10^4^ to 5 × 10^6^ Da.^5^ Glucose, fucose, arabinose, xylose, mannose and galactose are detected in PCP. β‐Glucan is the major PCP with β‐(1→3)‐linked glucose backbone and β‐(1→6)‐linked glucose side chains as shown in Figure 2.^3^, ^6^ The β‐glucan from *Poria cocos* has poor water solubility but decent anticancer activity.^7^ Chihara et al removed the β‐(1→6) glucose in the β‐glucan of PCP by periodate oxidation and Smith degradation. The derivative is named “pachymaran,” which exhibits better anti‐S‐180 tumour activities.^8^ Hamuro et al further improved the water solubility issue of pachymaran by chemical carboxymethylation. The carboxymethylated pachymaran (CMP) has enhanced antitumour activity compared to that of pachymaran.^9^ Subsequently, different chemical modifications, such as sulfation,^10^ carboxymethylation plus sulfation,^11^ methylation, hydroxyethylation and hydroxpropylation, have been conducted and different types of modified pachymarans are reported.^12^ In general, these chemical modified pachymaran derivatives are water‐soluble and show improved bioactivities.

### 1.2 PCP‐based drug in China

PCP‐based product named “compound polysaccharide oral solution” was developed in the 1970s and was very popular with consumers as health supplement products. In 2006, “Polysaccharidum of *Poria cocos* oral solution” was developed by Hunan Butian pharmaceutical company of China and was granted a Chinese patent (200610163425‐X). The major component (95%) in the patented product is CMP. The water solubility of CMP allows 98% of the components to be absorbed through the human digestive track. In 2015, “Polysaccharidum of *Poria cocos* oral solution” was approved by Chinese Food and Drug Administration with a certified drug number B20050015 for treating multiple types of cancers, hepatitis and other diseases alone or during chemo‐ or radiation therapy for patients with cancer.

### 1.3 The approach for the literature searching

**Figure 3:**
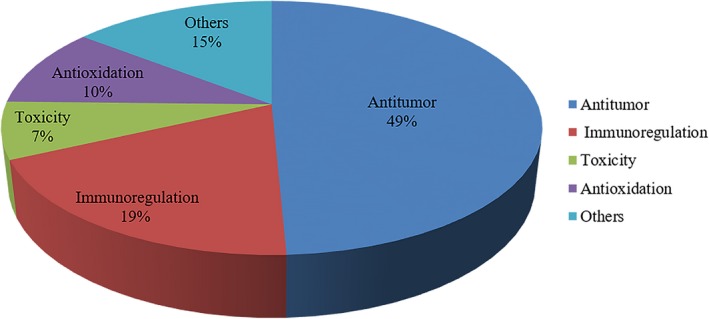
Pharmacological activities of *Poria cocos* polysaccharides (PCPs). Sixty‐six articles related to pharmacological activities of PCPs are summarized. Thirty‐eight per cent of studies are about antitumour activities of PCPs. Twelve per cent of studies are about antitumour mechanisms. Studies on immunoregulation, antioxidant and toxicity account for 19%, 10% and 7%, respectively. Fourteen per cent of pharmacological activity studies of PCPs are defined as “others” that are further explained in Figure 4

**Figure 4:**
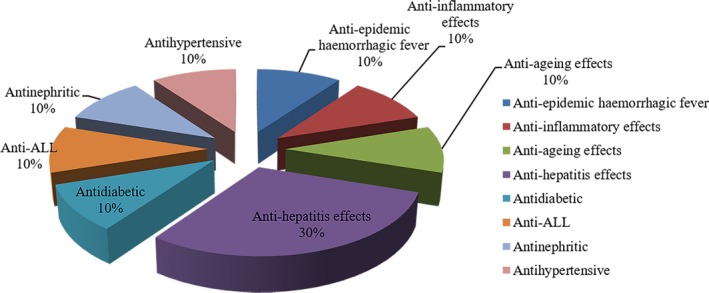
Other pharmacological activities of PCPs. Antihepatitis effects: 30%; antidiabetic effects: 10%; anti‐epidemic haemorrhagic fever: 10%; anti‐ageing effects: 10%; anti‐inflammatory effects: 10%; and anti‐acute lymphoblastic leukaemia (ALL): 10%

In this article, a total of 72 publications related to different kinds of *Poria cocos* polysaccharides (PCPs), pachymaran, the derivatives and the biochemical/preclinical/clinical studies up to date were identified through searching PubMed, VIP (Chongqing VIP Chinese Scientific Journals Database), CNKI (China National Knowledge Infrastructure) and Wanfang database. The reported biological and pharmacological activities of PCP are classified based on these publications and shown in Figures 3 and 4. The pharmacological and other biological functions and possible molecular mechanisms shown in Figures 3 and 4 will be the major topics discussed in this article. Moreover, the data that show PCPs can overcome immunosuppression and adverse reactions associated with radiation therapy and chemotherapy^13^ will also be presented and discussed.

## 2 PHARMACOLOGICAL ACTIVITIES OF PCP

### 2.1 Antitumour

Parallel to other reported polysaccharides from fungi,^14^, ^15^, ^16^ PCP and its derivatives have more impressive anticancer cell proliferation activities in vivo than in vitro when the same PCP samples are tested both in cancer cell lines and in cancer cell‐injected animal models. The in vitro anticancer cell proliferation effects of PCPs are summarized in Table 1, whereas the in vivo antitumour growth effects of PCPs are summarized in Table 2 from 15 independent studies.^17^, ^18^, ^19^, ^20^, ^21^, ^22^, ^23^, ^24^, ^25^, ^26^, ^27^, ^28^, ^29^, ^30^

**Table 1:** Abbreviation list

Abbreviation	Full name	Abbreviation	Full name
ALL	Acute lymphoblastic leukaemia	LCT	Lymphocytes transformation
ACV	Acyclovir	LLC	Lewis lung carcinoma
Bcl‐2	B‐cell lymphoma‐2	‐L	Low dosage
Bcap‐37	Breast carcinoma cells	‐M	Medium dosage
Bax	Bcl‐2 Assaciated X protein	MPCP	Methylated *poria cocos* polysaccharide
BHT	Butylated hydroxytoluene	MAO	Monoamine oxidase
CMP	Carboxymethylated pachymaran	MDA	Malondialdehyde
CTX	Cyclophosphamide	NS	Normal saline
CA	Cortisone acetate	NK	Natural killer cell
CP	Hericium erinaceus polysaccharide+Lentinan+Pachymaran	OH	Hydroxyl free radical
CPABM	Agaricus blazei murill polysaccharide+Lentinan+CMP	PCP	*Poria cocos* polysaccharide
DAG	Dianhydrogalactitol	PCP1	Polysaccharide extracted using 0.9% NaCI
DXM	Dexamethasone	PCP2	Polysaccharide extracted using hot water
DPPH	1,1‐Diphenyl‐2‐picrylhydrazyl radical 2,2‐Diphenyl‐1‐(2,4,6‐trinitrophenyl)hydrazyl	PCP3	Polysaccharide extracted using 0.5 mol/L NaOH
EC50	50% effective concentration	PCP4	Polysaccharide extracted using 88% formic acid
Ea	Active erythrocyte rosettle test	PTPP	Phosphorylation of protein tyrosine phosphatase
Et	Total erythrocyte rosettle test	PT	Pachymaran and triterpens
EAC	Ehrlich ascites carcinoma cells	PS	Pachymaran (Sulphated)
FP	Ferulic acid pachymaran	Pt	Cisplatin
HBsAg	Hepatitis B surface antigen	PAP	Acidic pachymaran
HBeAg	Hepatitis Be Antigen	SGC‐7901	Gastric carcinoma cells
HE‐PCP	Hydroxyethylated *poria cocos* polysaccharide	SOD	Superoxide dismutase
HP‐PCP	Hydroxypropylated *poria cocos* polysaccharide	SGPT	Serum glutamic pyruvic transaminase
H_22_	H_22_ hepatoma	TNF	Tumour necrosis factor
HPBL	Human peripheral blood lymphocyte	TPK	Tyrosine protein kinase
‐H	High dosage	U_‐14_	U‐14 ascitic fluid tumour cells
IC50	Half maximal inhibitory concentration	Vc	Vitamin C
IFN‐γ	Interferon‐γ	WPS	Polysaccharide extracted using NaOH‐HAc
KSC	Kappa‐selenocarrageenan	5‐Fu	5‐fluoro‐2,4(1H,3H) pyrimidinedione
LH	Levamisole hydrochloride		
Lewis	Lung carcinoma cell		

**Table 2:** Inhibition rates of PCPs on different cancer cells

Cell types	Groups	Cancer inhibition rates (%)	*P*‐value	References
SGC‐7901 (human)	Distilled water	0		17
	5‐Fu‐L	34.98		
	5‐Fu‐M	69.92		
	5‐Fu‐H	85.05		
	PCP2‐L	60.09		
	PCP2‐M	90.04		
	PCP2‐H	96.10		
Bcap‐37 (human)	5‐Fu‐L	35.41		
	5‐Fu‐M	61.04		
	5‐Fu‐L	88.72		
	PCP2‐L	42.48		
	PCP2‐M	85.09		
	PCP2‐H	88.54		
HepG2 (human)	PAP1	14.22 ± 1.06		4
	PAP2	16.65 ± 3.01		
	PAP3	6.94 ± 2.08		
	PAP4	15.98 ± 4.16		
	PAP5	16.71 ± 1.72		
	PAP6	16.02 ± 2.65		
	PAP7	59.76 ± 5.47	*P *< .05	
	PAP8	23.45 ± 1.31	*P *< .05	
	PAP9	78.67 ± 1.68	*P* < .01	
	PAP10	82.92 ± 2.8	*P* < .01	
K562 (human)	NS	0		30
	PS‐L	16.95 ± 5.16		
	PS‐M	27.80 ± 3.57		
	PS‐H	52.95 ± 1.2		

PCPs possess decent anticancer cell proliferation effect in vitro, which is measured in cell cultures where different tumour cell lines derived from human or mouse tumours have been tested, such as SGC‐7901 (human), Bcap‐37 (human), HepG2 (human) and K562 (human) cells (Table 2). For example, the inhibition rate for SGC‐7901 cells is 96% compared to that of 88% for Bcap‐37 cells.^17^ As shown in Table 2, the inhibition rates of PCP or its derivatives on the proliferation of cancer cells are largely concentration‐dependent, which resembles the control drug 5‐fluoro‐2,4(1H,3H) pyrimidinedione or 5‐Fu.

In contrast, in cancer cell‐injected animal models (Table 3), the inhibition rates of PCP or its derivatives on tumour growth are only partially concentration‐dependent in that within a certain range, the higher the concentrations, the higher the inhibition rates are. But beyond the range, the inhibition rates will drop. For example, the experiment conducted by Cheng et al^18^ showed that the inhibition rates of cancer cell growth are 0, 69%, 87%, 92% and 89%, respectively, with increasing CMP concentrations.

**Table 3:** Tumour inhibition rates of PCPs in animal models

Models	Administration route	Cell types	Groups	Tumour weight (g)	*P*‐value	Tumour inhibition rates (%)	*P*‐value	References
ICR mice	Intragastric administration	S_‐180_(mouse)	Distilled water	2.742 ± 0.378		0		20
			5‐Fu	1.341 ± 0.135	*P* < .001	45.73		
			CMP‐L	1.972 ± 0.399	*P* < .05	2.23		
			CMP‐H	1.675 ± 0.412	*P* < .01	32.22		
			5‐Fu+CMP‐L	1.413 ± 0.394	*P* < .001	42.83		
			5‐Fu+CMP‐H	1.283 ± 0.483	*P* < .001	48.11		
ICR/JCL mice	Intraperitoneal injection	U_‐14_ (mouse)	NS	1.385 ± 0.101		0		18
			CMP (30 mg/kg)	0.433 ± 0.105	*P* < .01	68.7		
			CMP (120 mg/kg()	0.182 ± 0.121	*P* < .01	86.9		
			CMP (180 mg/kg)	0.108 ± 0.053	*P* < .01	92.2		
			CMP (360 mg/kg)	0.151 ± 0.113	*P* < .01	89.1		
Kunming mice	Intraperitoneal injection	S_‐180_ (mouse)	NS	1.23 ± 0.11		0		21
			CTX	0.49 ± 0.07	*P* < .01	60.21		
			WPS‐L	0.99 ± 0.10		19.53		
			WPS‐M	0.89 ± 0.12	*P* < .01	28.01		
			WPS‐H	0.72 ± 0.08	*P* < .01	43.94		
			WPS1‐L	0.98 ± 0.13		2.32		
			WPS1‐M	0.96 ± 0.06	*P* < .1	22.45		
			WPS1‐H	0.70 ± 0.07	*P* < .01	41.57		
			WPS2‐L	1.09 ± 0.08		11.45		
			WPS2‐M	0.98 ± 0.10	*P* < .1	2.32		
			WPS2‐H	0.74 ± 0.04	*P* < .01	39.81		
Kunming mice	Intragastric administration	S_‐180_ (mouse)	NS	0.753 ± 0.191		0		22
			CTX	0.155 ± 0.091	*P* < .05	79		
			PCP‐L	0.437 ± 0.117	*P* < .05	42		
			PCP‐M	0.482 ± 0.105	*P* < .05	36		
			PCP‐H	0.527 ± 0.152	*P* < .05	30		
BALB/c mice	Intraperitoneal injection	S_‐180_ (mouse)	PBS	1.61 ± 0.32		0		19
			5‐Fu	0.76 ± 0.16		52.76	*P* < .01	
			PCP3‐II‐L	1.56 ± 0.42		2.46		
			PCP3‐II‐H	1.57 ± 0.66		3.02		
			PS‐L	1.39 ± 0.27		13.88		
			PS‐H	1.21 ± 0.41		34.63	*P* < .05	
			CMP‐L	1.23 ± 0.48		23.45	*P* < .05	
			CMP‐H	1.04 ± 0.20		35.27	*P* < .01	
			MPCP3‐II‐L	1.34 ± 0.46		16.65		
			MPCP3‐II‐H	1.22 ± 0.38		24.48	*P* < .05	
			HE‐PCP3‐II‐L	1.63 ± 0.36				
			HE‐PCP3‐II‐H	1.29 ± 0.29		20.20		
			HP‐PCP3‐II‐L	1.45 ± 0.27		10.00		
			HP‐PCP3‐II‐H	1.37 ± 0.48		14.88		
			PBS	1.40 ± 0.32		0		
			5‐Fu	0.76 ± 0.27		46.0	*P* < .01	
			PS‐2‐L	1.09 ± 0.26		22.32		
			PS‐2‐H	0.90 ± 0.36		35.71	*P* < .05	
			PS‐4‐L	0.89 ± 0.18		36.51	*P* < .05	
			PS‐4‐H	0.97 ± 0.36		30.95		
			PS‐5‐L	0.91 ± 0.34		34.92	*P* < .05	
			PS‐5‐H	0.86 ± 0.26		38.39	*P* < .05	
			PS‐6‐L	1.14 ± 0.12		18.25		
			PS‐6‐H	1.16 ± 0.39		17.35		
			PS‐9‐L	0.92 ± 0.29		33.93	*P* < .05	
			PS‐9‐H	0.93 ± 0.38		33.33	*P* < .05	
BalB/c mice	Intraperitoneal injection	S_‐180_ (mouse)	PBS	1.407 ± 0.32		0		23
			5‐Fu	0.867 ± 0.26		46	*P* < .01	
			PS	0.767 ± 0.27		38.39	*P* < .05	
ICR/JCL mice	Intragastric administration	S_‐180_ (mouse)	NS	1.989 ± 0.594		0		24
			5‐Fu	0.363 ± 0.286	*P* < .01	81.7		
			CMP‐L	1.282 ± 0.166	*P* < .05	35.5		
			CMP‐M	1.159 ± 0.126	*P* < .05	41.6		
			CMP‐H	0.963 ± 0.364	*P* < .05	51.6		
	Intravenous injection	S_‐180_ (mouse)	NS	3.425 ± 0.958		0		
			5‐Fu	0.323 ± 0.261	*P* < .01	90.6		
			CMP‐L	2.106 ± 1.037	*P* < .05	38.5		
			CMP‐M	2.294 ± 1.037	*P* < .05	32		
			CMP‐H	2.049 ± 0.752	*P* < .05	40.2		
	Intragastric administration	H_22_ (mouse)	NS	1.721 ± 0.571		0		
			5‐Fu	0.269 ± 0.230	*P* < .01	84.65		
			CMP‐L	0.760 ± 0.470	*P* < .05	55.58		
			CMP‐M	1.044 ± 0.438	*P* < .05	39.03		
			CMP‐H	0.644 ± 0.438	*P* < .01	62.24		
ICR/JCL mice	Intraperitoneal injection	U_‐14_ (mouse)	NS	1.225 ± 0.122		0		25
			CMP (25 mg/kg)	0.253 ± 0.11	*P* < .01	79.4		
			CMP (50 mg/kg)	0.26 ± 0.137	*P* < .01	78.8		
			CMP (100 mg/kg)	0.089 ± 0.003	*P* < .01	92.7		
			CMP (500 mg/kg)	0.30 ± 0.095	*P* < .01	75.5		
	Intravenous injection	S_‐180_ (mouse)	NS	3.431 ± 1.136		0		
			CMP‐L	2.237 ± 0.977	*P* < .05	34.8		
			CMP‐M	2.141 ± 0.969	*P* < .05	37.6		
			CMP‐H	1.339 ± 0.683	*P* < .01	61		
	Intravenous injection	H_22_ (mouse)	NS	2.167 ± 0.812		0		
			CMP‐L	1.732 ± 0.988		20.1		
			CMP‐M	1.372 ± 0.673	*P* < .05	36.7		
			CMP‐H	1.485 ± 0.931	*P* < .05	31.5		
NIH mice	Intragastric administration	S_‐180_ (mouse)	NS	1.34 ± 0.32		0		26
			5‐Fu	0.54 ± 0.43	*P* < .001	59.7	*P* < .01	
			CMP‐L	0.48 ± 0.34	*P* < .001	64.18	*P* < .01	
			CMP‐M	0.70 ± 0.36	*P* < .001	47.76	*P* < .05	
			CMP‐H	0.51 ± 0.53	*P* < .001	61.94	*P* < .01	
		S_‐180_ (mouse)	NS	1.30 ± 0.22		0		
			5‐Fu	0.67 ± 0.14	*P* < .001	48.46	*P* < .05	
			CMP‐L	0.54 ± 0.12	*P* < .001	58.46	*P* < .01	
			CMP‐M	0.78 ± 0.14	*P* < .001	40.00	*P* < .05	
			CMP‐H	0.74 ± 0.16	*P* < .001	43.08	*P* < .05	
		EAC(mouse)	NS	1.06 ± 0.16		0		
			5‐Fu	0.45 ± 0.16	*P* < .001	57.55	*P* < .01	
			CMP‐L	0.43 ± 0.18	*P* < .001	59.43	*P* < .001	
			CMP‐H	0.54 ± 0.21	*P* < .001	49.06	*P* < .05	
		EAC(mouse)	NS	1.02 ± 0.15		0		
			5‐Fu	0.41 ± 0.19	*P* < .001	59.80	*P* < .01	
			CMP‐L	0.51 ± 0.28	*P* < .001	50.00	*P* < .01	
			CMP‐H	0.62 ± 0.25	*P* < .001	39.22	*P* < .05	
ICR/ICJ mice	Intragastric administration	S_‐180_ (mouse)	NS	2.49 ± 0.42		0		28
			PCP‐L	1.29 ± 0.28	*P* < .01	48.1		
			PCP‐M	1.46 ± 0.46	*P* < .01	41.37		
			PCP‐H	2.25 ± 0.67		.96		
CFW mice	Intraperitoneal injection	S_‐180_ (mouse)	NS	5.1 ± 0.9		0		29
			PCP‐H	2.9 ± 1.0		43.1	*P* < .01	
			PCP‐M	3.1 ± 1.1		39.2	*P* < .01	
			PCP‐L	3.2 ± 1.7		37.3	*P* < .01	
Rats	Intraperitoneal injection	S_‐180_ (mouse)	NS	10.2 ± 2.6		0		27
			PCP	5.77 ± 2.7		39.9	*P* < .01	
			PS	6.10 ± 3.0		43.2	*P* < .01	

The inhibition of tumour growth in vivo is measured either by the reduced tumour weight or by ultrasound compared to controls in different animal models and presented as tumour inhibition rates (%). The controls include blank and positive controls where the chemotherapeutic drug, such as 5‐fluoro‐2, 4 (1H, 3H) pyrimidinedione (5‐Fu), is used. PCPs have potent antitumour activities in different animal tumour models when compared to blank or 5‐Fu controls (Table 3). When PCP is modified with different chemical groups, such as carboxymethyl, sulphate, methyl, hydroxypropyl and hydroxyethyl, the derivatives exhibit better inhibition rates. For example, Wang et al extracted six polysaccharides from the fresh sclerotium of *Poria cocos* using different solvents sequentially, including PCP1 (0.9% NaCl), PCP2 (hot water), PCP3‐I and PCP3‐II (0.5 mol/L NaOH), PCP4‐I and PCP4‐II (88% formic acid). They then modified PCP3‐II chemically and obtained five different PCP3‐II derivatives.^3^ They showed^19^ that all the derivatives inhibit tumour growth better than PCP3‐II with the inhibiting rates increased by 36% and 35% for sulphated and carboxymethyled derivatives, respectively, and by 24%, 15% and 20% for methylated, hydroxypropylated and hydroxyethylated derivatives, respectively. They further demonstrated that the increased degree of chemical modifications and the increased molecular weight in the derivatives correlate with better inhibiting rates in vivo.

Furthermore, the curing effects can be further enhanced when PCPs are combined with chemotherapeutic drugs in different animal models. In addition, the combined therapy also reduces the adverse effects associated with chemotherapeutic drugs (Table 4). For example, Liu et al^31^ Tang et al^32^ and Chen et al^29^ reported that PCPs exhibit better tumour inhibition rates when it is used with other chemotherapeutic drugs. For instance, inhibition rates were 46% when PCP is used with 5‐Fu compared with the inhibition rates of 41% when 5‐Fu is used alone.^29^

**Table 4:** Tumour inhibition rates of PCPs ± chemotherapy on mice

Models	Administration routs	Cell types	Groups	Tumour weight(g)	*P*‐value	Tumour inhibition rates (%)	*P*‐value	References
Kunming mice	Intragastric administration	S_‐180_ (mouse)	NS	3.01 ± 0.38		0		31
			CTX	0.84 ± 0.21	*P* < .01	72.1		
			CPABM‐L	2.10 ± 0.28	*P* < .01	30.2		
			CPABM‐M	1.43 ± 0.24	*P* < .01	52.5		
			CPABM‐H	2.69 ± 0.32		10.6		
			CPABM+CTX‐L	0.52 ± 0.16	*P* < .01	82.7		
			CPABM+CTX‐M	0.50 ± 0.19	*P* < .01	83.4		
			CPABM+CTX‐H	1.03 ± 0.31	*P* < .01	65.8		
Kunming and NIH mice	Intragastric administration	S_‐180_ (mouse)	NS	1.89 ± 0.24		0		32
			CP‐L	1.18 ± 0.29	*P* < .05	37.73 ± 13.11		
			CP‐M	1.03 ± 0.36	*P* < .05	44.74 ± 19.33		
			CP‐H	0.98 ± 0.26	*P* < .05	48.34 ± 13.08		
CFW mice	Intraperitoneal injection	S_‐180_ (mouse)	NS	1.4 ± 0.6		0		29
			5‐Fu	0.7 ± 0.2		50	*P* < .05	
			CTX	1.1 ± 0.4		21.4		
			DAG	0.9 ± 0.4		35.7	*P* < .05	
			PCP	1.0 ± 0.4		28.6		
			PCP+5‐Fu	0.9 ± 0.3		35.7	*P* < .05	
			PCP+CTX	0.8 ± 0.3		42.9	*P* < .05	
			PCP+DAG	0.8 ± 0.3		42.9	*P* < .05	

### 2.2 Antitumour mechanisms

**Figure 5:**
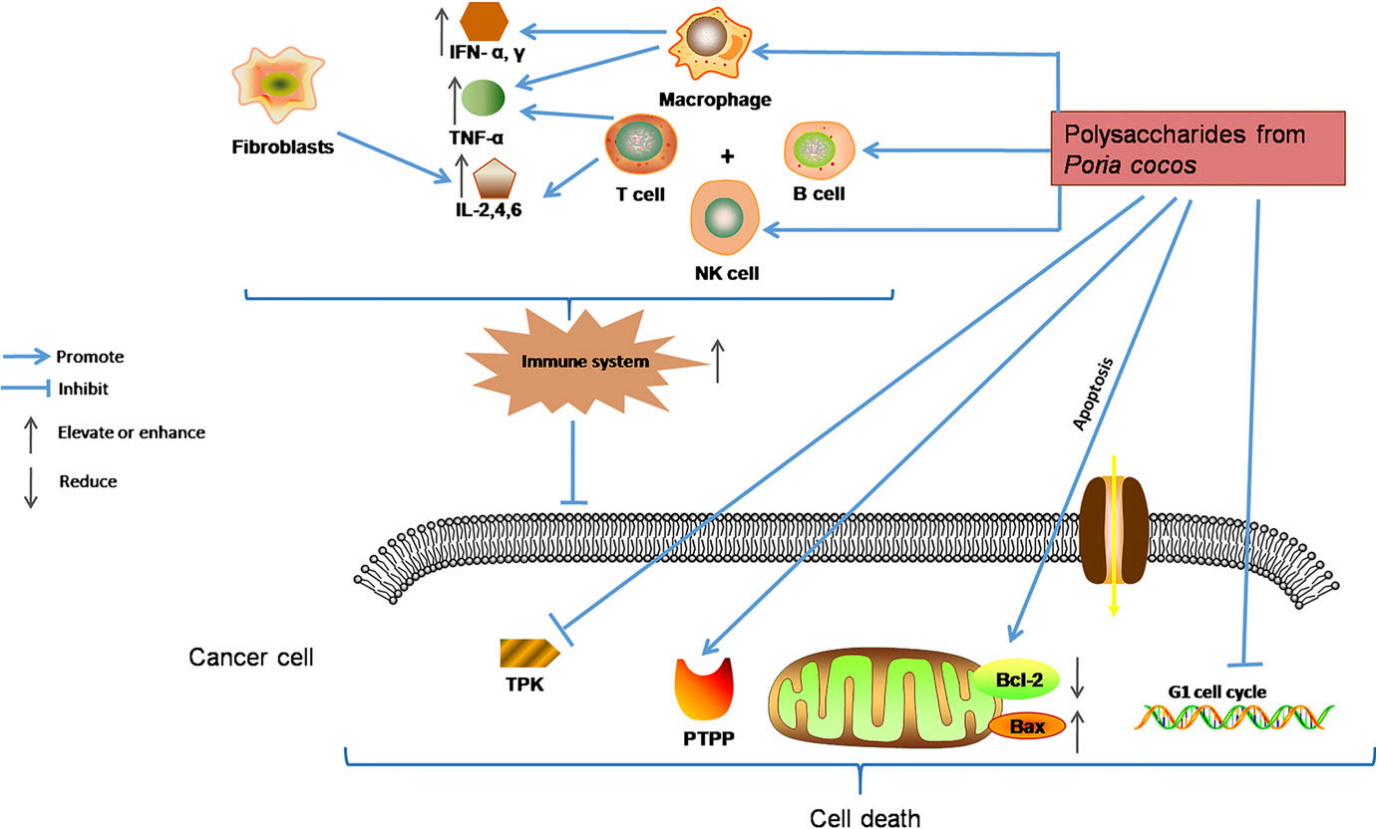
Possible antitumour mechanisms of PCPs. PCPs exert their antitumour activity via assisting the host to overcome adverse biological stresses, to assist the host to enhance the lethality of macrophages, T cells, B cells and NK cells by releasing cytokines to increase immunity, and to promote the apoptosis of tumour cells directly by up‐regulating the expression of apoptosis‐related genes

PCPs exert their antitumour activity via assisting the host to overcome adverse biological stresses, to increase immunity against the tumours and to promote the apoptosis of tumour cells directly. The possible mechanisms reported so far are summarized in Table 5A‐D and Figure 5, 33, 34, 35, 36, 37, 38 and discussed below.

**Table 5:** (A) Antitumour mechanisms of PCP: impact on expression rates of Fas, Bcl‐2 and Bax, the ratio of lymphocyte, killing activity of NK cell, IFN‐γ, TNF, phagocytic ability and IL‐2 (B) Antitumour mechanisms of PCP: impact on IFN‐γ, TNF, phagocytic ability, IL‐6, TPK in cytoplasm and cytomembrane, PTPP in cytoplasm and cytomembrane (C) Antitumour mechanisms of PCP: impact on phagocytic ability, thymus index, spleen index and IFN‐r (D) Antitumour mechanisms of PCP: impact on haemolysin, IL‐4, IgA in serum, IgG in serum and IgM in serum

Models	Administration routs	Cell types	Groups	Expression rates (%)			The ratio of lymphocyte (%)	Killing activity of NK cell (%)	IFN‐γ (IU/mL)	TNF (ug/mL)	Phagocytic ability (%)	IL‐2 (IU)	References
				Fas	Bcl‐2	Bax							
(A)													
BALB/c mice	Intraperitoneal injection	S_‐180_	PBS	4.12	47.96	2.45							33
			PS	64.99	17.97	48.05							
BALB/c mice	Intraperitoneal injection	H_22_	NS		46.52	45.38				0.08 (TNF‐α)			34
			CMP‐L		52.55	52.64				0.128 (TNF‐α)			
			CMP‐M		62.39	61.88				0.134 (TNF‐α)			
			CMP‐H		56.15	54.45				0.135 (TNF‐α)			
NIH mice	Intragastric administration	EAC	NS					12.01		8.05 × 10^^5^			26
			5‐Fu					16.01		12.28 **×** 10^^5^			
			CMP‐L					14.82		8.5 × 10^^5^			
			CMP‐H					15.56		9.69 × 10^^5^			
			NS									24.92	
			NS+CTX									13.01	
			CMP‐L+CTX									19.52	
			CMP‐H+CTX									21.67	
CFW mice	Intraperitoneal injection	S_‐180_	NS								9.3		29
			PCP‐L								21.9		
			PCP‐H								24.5		

Models	Administration routs	Cell types	Groups	IFN‐γ (IU/mL)	TNF (ug/mL)	Phagocytic ability (%)	IL‐6 (IU)	TPK in cytoplasm (min^−1^× μn^−1^)	TPK in cytomembrane (min^−1^ × μg^−1^)	PTPP in cytoplasm (min^−1^ × μg^−1^)	PTPP in cytomembrane (min^−1^ × μg^−1^)	References
(B)												
ICR/ICJ mice	Subcutaneous injection	LLC	NS			24.44						36
			CMP			59.26						
		S_‐180_	NS			26.88						
			CMP			45.92						
Cancer cells		HPBL	NS		5.0		1299.3					35
			CMP‐L		22.2		1356.3					
			CMP‐M		41.9		1837.4					
			CMP‐H		48.3		1880.6					
Cancer cells		HL‐60	Control					105.1	197.8	1.93	0.97	37
			PCP					62.5	158.1	6.08	3.14	
Cancer cells		HPBL	Control	3400	315 (U/mL)		4232					38
			CMP	6216	450 (U/mL)		6478					

Models	Administration routs	Groups	Phagocytic ability (%)	Thymus index	Spleen index	IFN‐γ (pg/mL)	References
(C)							
Kunming mice	Intragastric administration	NS	45.5	2.19	2.10		42
		LH	67.83	2.73	2,92		
		PT‐L	58.83	2.72	2.85		
		PT‐M	70.17	2.74	2.93		
		PT‐H	69.67	2.73	2.91		
C57BL/6 mice	Intravenous injection	NS			1.15		44
		Pt			0.42		
		PCP‐L			1.1		
		PCP‐H			1.41		
Kunming mice	Intraperitoneal injection	NS		3.09			45
		DXM		1.11			
		PS+DXM		1.89			
		PS		2.84			
BALB/c mice	Intraperitoneal injection	NS				77.00	43
		CMP				248.33	
NIH mice	Intragastric administration	NS	58.36	47.14	73.00		26
		CTX	37.64	23.17	42.34		
		CMP‐L	48.36	27.91	43.55		
		CMP‐M	51.09	30.28	54.88		
		CMP‐H	55.18	36.73	58.69		
ICR/ICJ mice	Subcutaneous injection	NS	39.65				36
		CMP	59.26				
		NS	30.39				
		CA	18.86				
		CA+CMP	34.81				
		CMP	50.48				
		NS		24.75	56.84		
		CTX		22.25	45.7		
		CTX+CMP		19.08	45.57		
		CMP		33.82	69.87		

Models	Administration routs	Groups	Haemolysin (OD_450_)	IL‐4 (pg/mL)	IgA in serum (μg/mL)	IgG in serum (ng/mL)	IgM in serum (ng/mL)	References
(D)								
Kunming mice	Intragastric administration	NS			61.45	286.64	2.23	46
		LH			97.32	378.46	2.81	
		PCP (60%)‐L			103.67	376.19	2.78	
		PCP (60%)‐M			122.38	570.30	3.53	
		PCP (60%)‐H			139.65	706.79	4.58	
Kunming mice	Intragastric administration	NS	0.4081	67.8		852.1	655.4	47
		CTX	0.1891	43.8		585.5	302.4	
		CTX+PCP‐L	0.2630	56.6		773.3	465.2	
		CTX+PCP‐H	0.2949	64.1		822.5	504.1	
		PCP	0.4967	84.1		1071.2	655.4	

#### 2.2.1 Enhancing the innate immunity through activating the immune cells

Polysaccharides could activate effector immune cells, such as macrophages, lymphocytes and natural killer (NK) cells to activate the innate immune system to exert antitumour activity by accelerating the host's defence mechanisms.^33^ It is reported that the ratio of lymphocytes could increase to 62% in the treatment group compared with control group (47%)^34^ and phagocytic ability of macrophages could reach 59% compared with control group of 27%.^36^

#### 2.2.2 Increasing cytokine levels

TNF (tumour necrosis factor) secreted by macrophages and lymphocytes is used as a drug for tumour biotherapy. IL‐6 (interleukin‐6) secreted by T lymphocytes and fibroblasts improves the killing ability of NK cells. Miu et al and Chen et al found that carboxymethyl pachymaran could improve the levels of IFN‐γ, IL‐2, TNF and IL‐6.^35^, ^38^

#### 2.2.3 Stimulating the expression of apoptosis‐related genes

Bcl‐2 and Bax are members of the Bcl‐2 family, which are important for the regulation of apoptosis. The Bax/Bcl‐2 ratio determines the survival of cells. Meng reported that sulphated pachymaran could enhance the expression of apoptosis‐related genes Fas and Bax and reduce the expression of Bcl‐2 gene. The increased Bax/Bcl‐2 ratio is responsible for the apoptosis of S180 tumour cells in the mouse model.^33^ Zhang et al showed that WPS could inhibit the growth of human breast carcinoma MCF‐7 cells by inducing G1 arrest of the cell cycle and by elevating the Bax/Bcl‐2 ratio.^39^

#### 2.2.4 Regulating the activities of TPK and PTPP in cancer cells

TPK (tyrosine protein kinase) and PTPP (phosphotyrosine protein phosphatase) are two important enzymes controlling the growth, proliferation and differentiation of cells. Several studies reported that the activities of TPK and PTPP have changed significantly when the cells become cancerous, but PCP normalizes the activities of TPK and PTPP in cancer cells and slowdowns cancer cell growth.^37^

### 2.3 Immunomodulation

Studies showed that PCP could enhance host immune function and activate the immune response.^26^, ^36^, ^40^, ^41^, ^42^, ^43^, ^44^, ^45^, ^46^, ^47^ It is reported that PCPs could modulate their specific immune response via the activation of T cells.^40^ Pachymaran strongly enhances the generation of alloreactive cytotoxic T lymphocytes in vivo.^41^ Generally speaking, the strength of the immune function can be judged through three aspects: the capability of macrophage's phagocytosis, the cellular immune function by detecting the index of immune organs^42^, ^43^ and the humoural immunity function by measuring the production of antibodies and serum haemolysins. As shown in Table 5C,D, Zhang et al and Xu et al found that the capability of macrophage's phagocytosis, thymus index and spleen index have significantly improved by PCP.^26^, ^42^ Zhang et al and Peng et al reported that the levels of IgA, IgG and IgM in serum are increased and the levels of IFN‐γ and IL‐4 are also enhanced by PCPs.^46^, ^47^, ^48^

### 2.4 Antioxidation

Free radicals refer to the dissociative molecules, atoms or ions with unpaired electron that reacting rapidly with other substances. Under certain range, free radicals can help to eliminate microorganisms intruding into body or abnormal cells. However, if free radicals are excessively produced, they would attack the normal cells and tissues. Therefore, human body constantly produces and removes free radicals to maintain a dynamic balance. Studies showed that PCP has antioxidant activity by scavenging free radicals. As shown in Table 6,^49^, ^50^, ^51^, ^52^, ^53^ Li et al and Zhang et al found that PCP2 and FP could clear free radicals (O_2_^−^, ·OH and DPPH·) and the clearance rate of DPPH· could reach 93%.^51^, ^52^ Wang et al also found that carboxymethylated PCP has DPPH, O_2_^−^ and ·OH radical‐scavenging activity in vitro.^54^, ^55^ Superoxide dismutase (SOD) is an important antioxidant enzyme in organisms and Chen et al showed that CMP could increase the expression of SOD and reduce the amount of MAD (malondialdehyde).^53^

**Table 6:** Antioxidant effects of PCPs

Groups	Deoxidization (abs)	Antioxidation (abs)	Clearance rate (%)	EC50 (g/L)	MDA in serum (nmol/mL)	MDA in hepar (nmol/mg·pro)	SOD activity in serum (U/mL)	SOD activity in hepar (U/mg·pro)	IC50 (mg/mL)	References
BHT	0.912	0.406								49
PCP	0.490	0.546								
CMP				2.5 (OH)						50
Vc				0.2 (·OH)						
CMP				>1.5 (O_2_^−^)						
Vc				1.5 (O_2_^−^)						
PCP (8 mg/mL)			76.7 (·OH)							51
PCP (10 mg/mL)			59.3 (O_2_^−^)							
PCP (5 mg/mL)			93.4 (DPPH)							
FP (4 mg/mL)			58.72 (·OH)	2.5(·OH)						52
FP (4 mg/mL)			39.7 (DPPH·)							
CMP									2.57 (O_2_^−^)	53
									7.66 (·OH)	
									4.56 (H_2_O_2_)	
NS					12.38	40.54	9.12	187.56		
CMP‐L					12.05	31.75	9.87	190.52		
CMP‐M					11.52	28.53	10.25	204.78		
CMP‐H					8.74	24.62	12.67	224.63		

### 2.5 Other pharmacological activities

PCPs have other biological effects, such as anti‐inflammatory,^56^ anti‐ageing,^57^ antihepatitis,^58^, ^59^ antidiabetic,^60^ anti‐ALL (acute lymphoblastic leukaemia),^61^ anti‐nephritic^62^ and antihypertensive effects.^63^ These pharmacological activities are summarized in Table 7A,B. Hou et al observed that PCP reduces the size of granuloma.^56^ As an anti‐ageing reagent, PCPs enhance the activities of both T‐SOD and Cu‐SOD and reduce MAD and MAO (monoamine oxidase) activities.^57^ To understand the antihepatitis effects of CMP, it is found that CMP reduces the expression of HBsAg and HBeAg in a concentration‐dependent manner.^58^ Zheng et al reported that PCP could reduce blood glucose level and increase the weight of mice in a diabetic mouse model.^60^ Meanwhile, CMP significantly improves the survival rates of mice suffering acute lymphoblastic leukaemia or ALL.^61^

**Table 7:** (A) Other pharmacological effects of PCPs: anti‐inflammatory, anti‐ageing and antihepatitis effects (B) Other pharmacological effects of PCPs: antidiabetic and anti‐acute lymphoblastic leukaemia effects

Other effects	Groups	Swelling degree (mg)	Granuloma (mg/10 g)	T‐SOD	Cu‐SOD	MDA	MAO	Death time of swimming (min)	Inhibition of HBsAg (%)	Inhibition of HBeAg (%)	Inhibition of Anti‐HBc	Inhibition of SGPT	References
(A)													
Anti‐inflammatory effects	NS	6.2	47										56
	DXM	3.2	13										
	PCP‐L	3.6	36										
	PCP‐M	7.4	40										
	PCP‐H	8.5	38										
Anti‐ageing effects	Double distilled water			108	46	7.1	16	2.48					57
	PCP‐L			114	54	6.7	19	3.68					
	PCP‐M			125	60	6.5	16	4.93					
	PCP‐H			127	62	6.3	13	6.15					
Antihepatitis effects	ACV‐L								23.5	18.7			58
	ACV‐M								50.7	30.5			
	ACV‐H								61.4	51.1			
	CMP (1.5 g/L)								37.4	30.4			
	CMP (3.0 g/L)								46.3	47.0			
	CMP (6.0 g/L)								56.2	58.5			
	CMP (12.0 g/L)								71.8	65.3			
	Traditional Chinese medicine								22.3	36.3	28.5	32.3	59
	CMP‐induced IFN‐α+ Traditional Chinese medicine								52.1	95.4	75	80	

Other effects	Groups	The weight of mice after 30 days (g)	Blood glucose of mice after 30 days (mg %)	Life prolonging rates (%)	Expression rates of Bcl‐2	References
(B)						
Antidiabetic effects	Distilled water	163.8	19.72			60
	Melbine	168.64	11.35			
	PCP‐L	201.32	15.4			
	PCP‐H	213.58	15.16			
Effects on ALL mice	NS			0	18.4	61
	CTX			58.84	40.5	
	KSC			25.83	68.9	
	CMP			35.88	36.3	
	KSC+CMP			43.97	40.1	
	KSC+CMP+CTX			79.68	26.7	

IFN‐α was obtained from human peripheral blood lymphocytes treated with CMP.

## 3 CLINICAL EFFICACY OF PCP

Seven clinical studies on PCPs were found through literature search.^59^, ^64^, ^65^, ^66^, ^67^, ^68^ The clinical data are summarized in Table 8. Most of studies are related to the antitumour effects of CMP where these studies use IL‐2 or IFN‐α obtained from human peripheral blood lymphocytes induced by CMP in combination with chemotherapy or radiotherapy. Sheng et al reported that the total effective rate could reach 97% when CMP‐induced IL‐2 is combined with chemotherapy during cancer treatment compared to that of 27% by chemotherapy alone.^65^ The effectiveness is defined as improving the symptoms of the disease by increasing appetite, elevating the levels of cAMP in blood circulation, regulating the ratio of cAMP/cGMP, protecting and restoring damaged liver and reducing the side effects of chemotherapy.^66^ In treating epidemic haemorrhagic fever, cure rate could reach 100% when CMP‐induced IFN‐α is combined with normal therapy compared to that of 63% with normal therapy alone.^64^ Chen et al also found that the total effective rate could reach 90% during hepatitis treatment.^66^ The changes of immune functions are measured with Et (total erythrocyte rosettle test), Ea (active erythrocyte rosettle test) and LCT (lymphocytes transformation) during PCP treatment. These values are significantly increased compared to the control groups when PCP is combined with chemotherapy^67^ (Table 8).

**Table 8:** PCP‐related clinical studies

Diseases types	Cases	Groups	Total effective rate (%)	NK cell activity (%)	Cure rate (%)	Et (%)	LCT (%)	Ea (%)	References
Antitumour effects	71	Mitomycin/cis‐platinum/pharmorubicin/5‐Fu	25.9						63
		Mitomycin/cis‐platinum/pharmorubicin/5‐Fu + CMP‐induced IL‐2	86.7						
	44	Mitomycin/cis‐platinum/doxorubicin/5‐Fu + CMP‐induced IL‐2	86.4						65
	37	^60^Co radiotherapy + CMP‐induced IL‐2	97.3						
	77	^60^Co radiotherapy + CMP‐induced IFN‐α	97.4						59
	25	Mitomycin/cis‐platinum/pharmorubicin/5‐Fu+ CMP‐induced IFN‐α	92						
Effects on epidemic haemorrhagic fever	128	Balanced salt solution	60		68.7				64
		Balanced salt solution+ IFN‐α	63		100				
Antihepatitis effects	35	CMP	88.57		54.28				66
	30	CMP	90		36.67				
	30	CMP	90		30				
	60	Mitomycin/cis‐platinum/pharmorubicin/5‐Fu				30.7	38.7	20.5	67
		Mitomycin/cis‐platinum/pharmorubicin/5‐Fu+ PCP				38.5	50.4	27.5	
	50	Control				47.4	46.2	23.3	68
		PCP				49.2	55.4	33.3	

IL‐2 and IFN‐α were obtained from human peripheral blood lymphocytes treated with CMP.

## 4 TOXICITY

CMP has very low toxicity. It is reported by Chen et al^69^ that the mice moves freely, no abnormal reaction is observed when CMP is used during standard acute toxicity test. No teratogenic effects are detected on rats when CMP is used in the teratogenic tests. No toxic reaction is witnessed when the dogs are continuously injected by intravenous injection reported by Ye et al^70^ They further demonstrated that blood pressure, heart rate, electrocardiogram and breathy of dogs are not affected after intravenously injecting either 400 mg/kg or 800 mg/kg CMP. In consistent, Chai et al^71^ reported that CMP has no adverse effects on mice as well. Wang et al showed that CMP could enhance the tumour inhibition rate of 5‐FU and decrease the liver injuries simultaneously caused by 5‐FU in CT26 tumour‐bearing mice.^72^

## 5 FUTURE PERSPECTIVES

In this article, the structure, pharmacological effects, clinical efficacy, immunobalancing molecular mechanism and toxicity of PCPs are summarized in Figures 1, 2, 3, 4, 5 and Tables 1, 2, 3, 4, 5, 6, 7, 8. The broad spectrum of therapeutic properties, relatively low toxicity and low costs make PCPs attractive immune therapeutics for treating not only different types of cancers but also hepatitis B and other diseases.

Both advantages and disadvantages of PCPs as drugs rely on their complicated polysaccharide structure‐dependent immune regulatory functions. Thus, there is a great need for clarifying the active ingredients in PCPs besides β‐glucan/pachymaran and their molecular targets responsible for their drug effects. In addition, how to standardize the quality of PCPs, especially the degree of chemical modifications of pachymaran derivatives and how to perform reliable pharmacokinetic studies of PCPs are some of important issues to be solved in near future to make use of PCPs for treating patients with cancer worldwide.

## CONFLICT OF INTEREST

The authors declare no conflict of interests. All grants and funding agencies play no role in the study design; in the collection, analysis and interpretation of data; in the writing of the manuscript; and in the decision to submit the manuscript for publication.
